# An Improved Neural Network Model Based on DenseNet for Fabric Texture Recognition

**DOI:** 10.3390/s24237758

**Published:** 2024-12-04

**Authors:** Li Tan, Qiang Fu, Jing Li

**Affiliations:** College of Science & Technology, Ningbo University, Ningbo 315300, China; 13511146910@163.com (L.T.); lijing3@nbu.edu.cn (J.L.)

**Keywords:** channel attention, deep learning, DenseNet, differentiated learning, fabric texture recognition

## Abstract

In modern knitted garment production, accurate identification of fabric texture is crucial for enabling automation and ensuring consistent quality control. Traditional manual recognition methods not only demand considerable human effort but also suffer from inefficiencies and are prone to subjective errors. Although machine learning-based approaches have made notable advancements, they typically rely on manual feature extraction. This dependency is time-consuming and often limits recognition accuracy. To address these limitations, this paper introduces a novel model, called the Differentiated Leaning Weighted DenseNet (DLW-DenseNet), which builds upon the DenseNet architecture. Specifically, DLW-DenseNet introduces a learnable weight mechanism that utilizes channel attention to enhance the selection of relevant channels. The proposed mechanism reduces information redundancy and expands the feature search space of the model. To maintain the effectiveness of channel selection in the later stages of training, DLW-DenseNet incorportes a differentiated learning strategy. By assigning distinct learning rates to the learnable weights, the model ensures continuous and efficient channel selection throughout the training process, thus facilitating effective model pruning. Furthermore, in response to the absence of publicly available datasets for fabric texture recognition, we construct a new dataset named KF9 (knitted fabric). Compared to the fabric recognition network based on the improved ResNet, the recognition accuracy has increased by five percentage points, achieving a higher recognition rate. Experimental results demonstrate that DLW-DenseNet significantly outperforms other representative methods in terms of recognition accuracy on the KF9 dataset.

## 1. Introduction

Textiles (Ningbo Cixing Co., Ltd., Ningbo, China) play a crucial role in the apparel industry, providing essential functions such as warmth, sun protection, wind resistance, and rainproofing, thereby supporting fundamental aspects of human life. As a significant sector of the global economy, the textile industry not only generates substantial employment but also drives economic growth. With technological advancements, functional fabrics have found widespread applications in medical, outdoor, and industrial fields. In fabric production, accurately identifying knit textures is a critical step prior to processing, as it directly influences the operation of knitting machines. Currently, fabric texture recognition relies primarily on manual inspection and magnification tools, which are prone to errors due to operator fatigue and subjective bias. Consequently, the development of automated fabric texture recognition systems has become an urgent need to enhance efficiency and ensure consistent product quality.

In recent years, textile texture recognition has garnered significant attention and achieved remarkable progress. Methods for identifying knitted fabric textures are typically divided into two categories: statistical classification based on texture features and deep learning classification based on databases. Texture-based statistical methods primarily analyze preprocessed images. For instance, Kuo et al. [1] and Pan et al. [2,3,4] applied backpropagation neural networks to classify knitted fabric textures, utilizing a white–black co-occurrence matrix to classify pre-identified patterns stored in a fabric pattern database. Additionally, Kuo and Kao [5] combined co-occurrence matrices with the CIE-Lab color model to extract texture features, followed by a self-organizing map network for fabric classification. However, the accuracy and performance of these methods heavily depend on the scale of the available databases. Fan et al. [6] proposed a method that combines K-means clustering, grayscale gradient accumulation, and Gabor filters for yarn segmentation, texture localization, and recognition. Despite its merits, this approach struggles with blurred textures and does not fully account for lighting variations during image capture. Li et al. [7] proposed a photometric differential analysis method, which employs histogram equalization and an adaptive Wiener filter to capture fabric texture information from multiple angles. Then, an adaptive grid model was utilized to divide images into sub-images for grayscale feature extraction. While this method demonstrates robustness in detecting intersections within woven textures, it is limited to simpler fabric texture types. The performance of texture-based statistical classification methods is ultimately constrained by the quality of handcrafted features. Furthermore, the limited availability of fabric image databases, coupled with factors like rotational variations and improper lighting, directly affects the extraction of texture features.

In contrast, deep learning methods trained on databases provide an end-to-end recognition system. However, current database-based deep learning methods for knitted fabric classification are quite limited. Most deep learning methods focus on model implementation without introducing significant optimizations. Although these models outperform texture-based statistical classification methods, they often lack the targeted enhancements necessary for handling specific challenges in fabric texture recognition.

To address these limitations, we propose a Differentiated Leaning Weighted DenseNet (DLW-DenseNet). This model integrates adjustable weights into the original DenseNet architecture, which enhances relevant features from informative channels while suppressing those from redundant of irrelevant channels, thereby improving recognition accuracy. Typically, a low learning rate is used to ensure model convergence, but this can render the impact of adjustable weights negligible. To counter this, we introduce a differential learning rate strategy, which increases the learning rate for the weighted layers. This facilitates pruning by amplifying the weights of useful channels and reducing the weights of redundant channels to near-zero. Our model demonstrates significant improvements in fabric texture recognition compared to other representative deep learning methods.

The key contributions of this paper are as follows:

1. A mechanism for adjustable weights is incorporated into DenseNet to enhance relevant features and suppress redundant or irrelevant ones, thereby improving model accuracy;
2. A differential learning rate strategy is employed to optimize weighted layers, resulting in effective pruning;
3. We collect and construct a dataset named KF9 (knitted fabric) for fabric texture recognition, and conduct a preliminary analysis;
4. Our model outperforms representative convolutional neural networks on the KF9 dataset as well as on the CIFAR-10 and CIFAR-100 benchmarks.

The remainder of this paper is organized as follows: Section 2 offers a comprehensive review of prior search on fabric texture recognition. Section 3 details the design and architecture of the proposed Differentiated Leaning Weighted DenseNet (DLW-DenseNet) model. In Section 4, we describe the creation and characteristics of the KF9 dataset, and Section 5 presents the experimental results and performance evaluation of the proposed model.

## 2. Related Work

Most traditional fabric recognition methods have relied on statistical classification based on texture and conventional machine learning approaches. For instance, Trunz et al. [8] introduced a method for identifying and locating stitch types by inferring the underlying mesh structure in knitted fabrics. Nevertheless, their experiments were confined to knit and polyester stitches, and their method, which belongs to reverse engineering fabric prototypes, analyzed only single images. Yildiz [9] explored dimensionality reduction using Principal Component Analysis for defect classification in velvet fabrics, with the K-Nearest Neighbor classifier outperforming Naive Bayes. However, this method overly emphasized binary patterns and failed to effectively address complex texture patterns. Li et al. [10] combined Local Binary Patterns with the Gray-Level Co-occurrence Matrix for feature extraction and utilized Support Vector Machines to classify fabric textures. Despite improving recognition accuracy compared to LBP or GLCM alone, it still relied on manual feature design and was not extended to non-woven and knitted fabrics. Guo et al. [11] introduced a Local Feature Similarity method to identify the repeat size of woven patterns, while Xiao et al. [12] used Fuzzy C-Means clustering to detect warp and weft crossing. However, most of these methods struggle to handle significant rotational variations, common in real-world applications. Overall, texture-based statistical methods remain dependent on handcrafted features, and their recognition performance is suboptimal.

In recent years, the rapid development of deep learning technologies has led to significant achievements in numerous fields, offering resolutions to practical problems in production processes such as channel modeling [13,14]. Increasingly, researchers have integrated machine vision with deep learning into intelligent manufacturing, applying these technologies to tasks like waste sorting [15] and defect localization [16]. Within the clothing industry, intelligent manufacturing, focusing on personalized design and customized garment features, has become increasingly prominent. Fabric texture recognition plays a critical role in ensuring garments meet customer expectations for structure, shape, patterns, and materials.

Neural network architectures have evolved significantly since the 1980s [17], with early cascade structures resembling modern dense networks. The global adoption of neural networks began with AlexNet [18], followed by VGGNet [19], ResNet [20], and DenseNet [21]. AlexNet introduced the dropout mechanism and was the first to harness GPUs for model training, significantly accelerating the process. VGGNet demonstrated the efficacy of small convolution kernels and deeper networks for improved performance, while ResNet introduced residual connections to prevent the vanishing gradient problem, enhancing model convergence. DenseNet built upon these innovations by facilitating feature reuse across layers, though it can lead to information redundancy as layers accumulate. In the domain of fabric defect detection, Liu et al. [22] proposed an optimized convolutional neural network method for fabric defect detection, which incorporates a visualization technique for complex textures. The experiment was conducted using a very limited number of photos, under very stringent conditions. Ouyang et al. [23] introduced a pioneering approach for fabric defect detection by combining paired potential activation layers in CNNs. This method effectively improved the accuracy of fabric defect recognition by leveraging statistical defect data, even when dealing with imbalanced datasets and complex features. In the domain of fabric texture classification, Iqbal Hussain et al. [24] used an improved version of the ResNet50 model for fabric structure classification. To mitigate the issue of overfitting, they added two fully connected layers, two batch normalization layers, and two dropout layers at the end. The inclusion of global average pooling and dropout layers effectively reduces the issue of overfitting. Akram et al. [25], on the other hand, modified the VGG16 model by adding two additional pooling layers, improving accuracy and reducing computation time with a smaller dataset.

However, these implementations lacked model-specific optimizations. To address this, we modified DenseNet by introducing a weighted module tailored to the characteristics of the KF9 dataset, which is relatively small with high inter-class similarity. This module assigns a learnable weight to each channel, significantly reducing information redundancy and expanding the model’s search space. To counter this, we introduce a differential learning strategy, assigning distinct learning rates to different model components. This strategy enhances channel selection, improves pruning efficiency, accelerates convergence, and increases overall model accuracy.

## 3. Method

### 3.1. DenseNet

In traditional convolutional neural networks (CNNs), the input to each layer is merely related to the output of the preceding layer. DenseNet, however, introduces an innovative connection mechanism by concatenating the feature maps of all preceding layers along the channel dimension, promoting feature reuse and improving information flow across the network.

**Figure 1:**
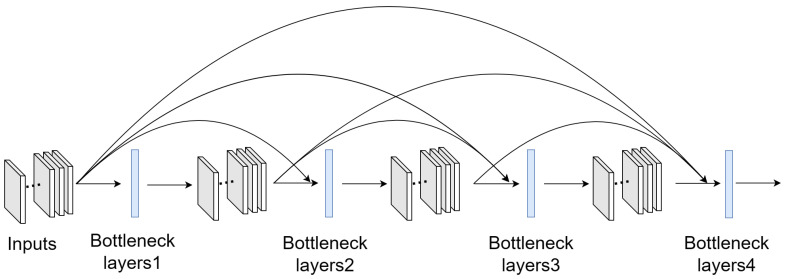
The original 4-layer DenseNet block.

Figure 1 illustrates the architecture of a dense block, where each layer consists of Batch Normalization (BN), a ReLU activation function, and a 3 × 3 convolution operation (BN-ReLU-3 × 3 conv). To facilitate feature reuse, each layer receives the feature maps from all previous layers as input, thereby enhancing the model’s ability to extract and refine complex features.

In a dense block, all convolution operations produce feature maps of the same spatial dimensions, enabling direct dense connections along the channel dimension. Except for the initial convolution, each subsequent convolution generates an equal number of feature channels, determined by the growth rate, k. A higher k increases the flow of information, thereby enhancing the network’s feature extraction capability. However, this also increases the computational complexity of the model.

As a result of the dense connectivity, particularly in the deeper layers of a dense block, the number of input features grows substantially. To mitigate this, a 1 × 1 convolution, referred to as the bottleneck layer, is introduced before each 3 × 3 convolution. This bottleneck layer, composed of BN, ReLU, and a 1 × 1 conv operation, reduces the number of input features, improving computational efficiency without compromising performance.

Within a dense block, feature map sizes remain constant, as no downsampling occurs. DenseNet, therefore, divides the network into multiple dense blocks, with transition layers between each block to perform downsampling. Each transition layer consists of a 1 × 1 convolution to adjust the number of channels, followed by a 2 × 2 average pooling layer, which reduces the spatial dimensions of the feature maps by itself.

### 3.2. Differentiated Leaning Weighted DenseNet

#### 3.2.1. Weighted Module

**Figure 2:**
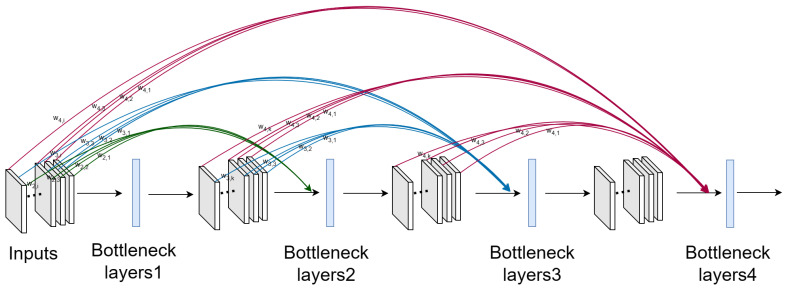
A 4-layer weighted DenseNet block. In this block, each feature channel is assigned a weight w, denoted by two subscripts. The first subscript indicates the layer to which the weight is applied, while the second subscript corresponds to the specific feature channel. For example, w_4,1_ represents the weight applied by the fourth layer to the first feature channel. For clarity, only the weights associated with cross-layer feature channels are illustrated, while the weights within non-cross-layer channels are omitted.

DenseNet encourages extensive feature reuse, allowing subsequent convolutional layers to utilize features extracted by earlier layers. However, as the network depth increases, this can lead to significant information redundancy. To mitigate this and enhance feature selection (as depicted in Figure 2), we incorporated a custom direct weighting module into the original DenseNet architecture. This module assigns a learnable weight coefficient to each input feature channel, enabling the model to dynamically adjust the contribution of each channel’s features according to the specific needs of the task. The model can opt to utilize all, part, or none of a given channel’s features. For instance, as inputs are passed into layer 1, the weighting module dynamically assigns a weight to each feature channel, allowing the model to control the contribution of each channel based on its relevance. Through this flexible weighting mechanism, the model effectively reduces redundant information and expands its feature search space, thereby improving overall performance and efficiency.

**Figure 3:**
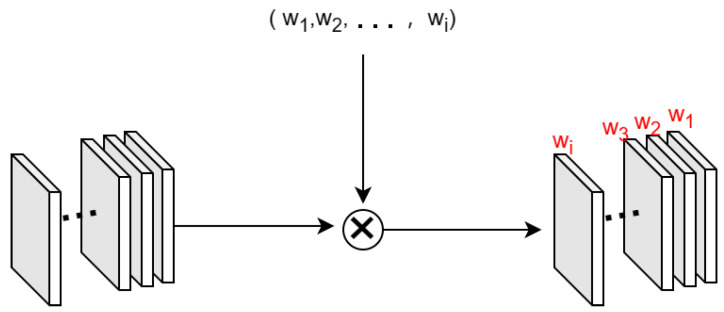
The process of input feature weighting involves multiplying each feature channel by its corresponding weight parameter, represented by the symbol ◯× .

As shown in Figure 3, the direct weighting module generates a set of learnable weight coefficients, with the number of coefficients corresponding to the number of input feature channels. Each coefficient is multiplied with its respective input feature channel, allowing the model to apply a learnable weight to each channel, thereby enabling fine-grained control over feature importance during the learning process.

After passing through the direct weighting module, each input feature channel is associated with a learnable weight.

(1) $$X_{i}=F_{i}\;\times \;\left(W_{in\_channels}\times \;X_{in\_features}\right)$$

Specifically, $F_{i}$ represents the nonlinear transformation of the current layer, $X_{in\_features}$ denotes the input features of the current layer, and $W_{in\_channels}$ refers to the learnable weight for each input channel in the current layer.

By optimizing these parameters through backpropagation and utilizing an appropriate optimizer, the model dynamically adjusts the weight coefficients associated with each feature channel. This enables the model to selectively emphasize or diminish features based on their relative importance, thereby enhancing its capacity to extract the most relevant information from each channel.

#### 3.2.2. Differential Learning Strategy

**Figure 4:**
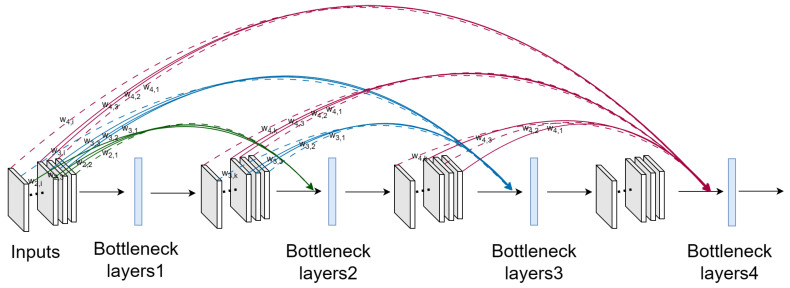
The 4-layer Weighted DenseNet block with the introduction of a differential learning strategy. The dashed lines indicate channels where the corresponding weights have been reduced to near zero during the learning process, effectively performing pruning.

However, due to the typically low overall learning rate—particularly in the later stages of training—the learnable weights of each channel tend to change very little, diminishing the model’s ability to effectively select important channels. To address this issue, we implement a differential learning strategy by assigning varying learning rates to different parts of the model (as shown in Figure 4). Specifically, during the experimental process, we markedly increased the learning rate associated with the learnable weights for each channel, relative to the learning rates of other parts of the model. This adjustment emphasized the importance of channel selection, enabling the model to prioritize critical channels or discard redundant and non-informative ones more effectively during iterative training. This strategy significantly enhances the model’s ability to focus on the most relevant channels within the input features. As a result, the weights of certain channels approach zero, achieving effective pruning, reducing redundancy, accelerating convergence, and improving model accuracy.

## 4. Knit Fabric Dataset

Fabric texture recognition currently depends heavily on skilled technicians, rendering the process both time-consuming and labor-intensive, while also susceptible to errors caused by fatigue, mental strain, and other human factors. With the rapid advancements in CNNs, computer vision applications in fabric recognition have made substantial progress. However, the performance of CNN models is largely contingent on the availability of large-scale, high-quality datasets. Given the critical importance of well-curated datasets for effective model training, and the absence of publicly available datasets for knitted fabric structures, we develop a custom dataset, KF9 (knit fabric), specifically designed to address this gap and support further advancements in fabric texture recognition.

### 4.1. Dataset Construction

#### 4.1.1. Data Collection

**Figure 5:**
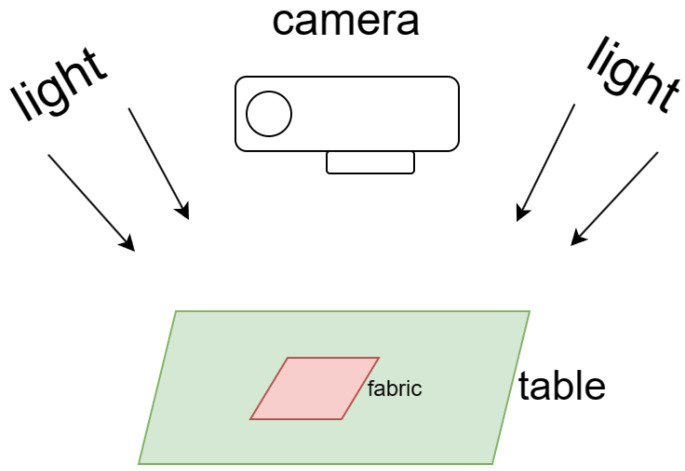
Photography setup, where “light” represents the strong, uniform lighting conditions. The “camera” refers to the high-resolution digital camera used for capturing detailed images, while the “table” serves as the platform for positioning the fabric samples. The “fabric” refers to the knitted fabric textures being photographed.

As shown in Figure 5, we employ a Fujifilm T30 digital camera equipped with a 40 mm macro lens in a dedicated photography studio to capture high-resolution images of fabric textures. Ensuring optimal lighting conditions and image clarity, we collect 523 representative knitted fabric samples from a professional textile factory specializing in knitted garments. Given that each fabric piece often contains multiple structural patterns, we capture a total of 2040 images from various sections of the fabrics, providing a comprehensive dataset for analysis.

#### 4.1.2. Data Processing

After obtaining the raw images of the fabric textures, we employ various data augmentation techniques to expand the dataset. These augmentation operations include vertical and horizontal flipping, scaling, brightness adjustments, shearing, translation, and rotation. Rotation, in particular, is crucial to account for posture variations during image capture. This augmentation process substantially increased the dataset size, which is beneficial for deep learning models that typically perform better with larger datasets.

By augmenting the data, we not only enrich the diversity of the training set but also improve the model’s ability to generalize, thereby reducing the risk of overfitting. Data augmentation functions similarly to regularization, enhancing model robustness without altering the original dataset. Following augmentation, we manually screen the dataset to remove any invalid or redundant images, ensuring the integrity and quality of the final dataset before categorization.

### 4.2. Dataset Statistics

**Figure 6:**
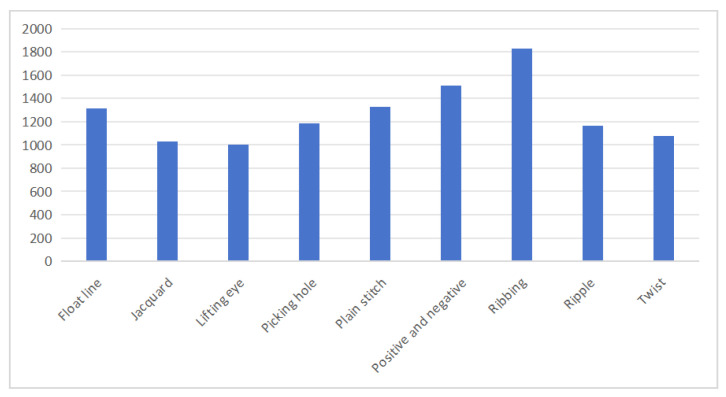
Class cardinality of different categories in the KF9 knitted fabric dataset.

**Figure 7:**
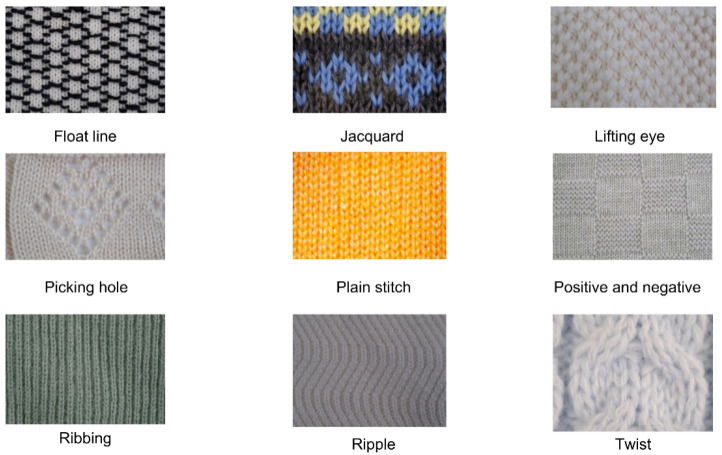
The legend corresponding to the nine categories of knitted fabric textures.

Following data augmentation, the KF9 knitted fabric dataset contains 13,259 samples distributed across nine categories: float line, jacquard, lifting eye, picking hole, plain stitch, positive and negative, ribbing, ripple, and twist patterns. Each category corresponds to a distinct, commonly used knitted fabric texture, as illustrated in Figure 6, with representative images provided in Figure 7. On average, each category contains slightly over 1000 samples, though the distribution varies. The ribbing category has the largest representation with 1828 images, while the drop stitch category contains the fewest, with 1003 images. All images were captured at a resolution of 1560 pixels wide by 1040 pixels high, yielding an aspect ratio of approximately 1.5:1, ensuring sufficient detail for accurate analysis of the fabric structures.

## 5. Experiments

To assess the effectiveness of the Differential Learning Weighted DenseNet (DLW-DenseNet), we conduct extensive experiments on both our custom KF9 knitted fabric dataset and two widely-used benchmark datasets, CIFAR-10 and CIFAR-100.

### 5.1. Datasets and Experimental Setup

#### 5.1.1. Datasets

We first evaluate the model’s performance using our self-constructed KF9 dataset, which comprises 13,259 images spanning nine fabric categories. Of these, 11,440 images are allocated to the training set, while 1819 images are reserved for testing. To further validate the model’s effectiveness, we conduct additional experiments on the publicly available CIFAR-10 and CIFAR-100 datasets. The CIFAR datasets consist of 32 × 32 pixel color images of natural scenes, with 50,000 images for training and 10,000 for testing. CIFAR-10 contains 10 categories, while CIFAR-100 includes 100 categories.

#### 5.1.2. Experimental Setup

The experiments are implemented using the PyTorch framework on a PC equipped with an Intel i7-12700K CPU, an Nvidia GeForce RTX 3090 GPU, and 32GB of RAM.

For the KF9 dataset, the learning rate is set to 0.001 and the Adam optimizer is utilized for training. Random cropping is employed as a data augmentation technique to enhance model generalization.

For the CIFAR-10 and CIFAR-100 datasets, we adopt the hyperparameter settings from the original DenseNet paper to ensure a fair comparison. Data augmentation techniques include random sampling and horizontal flipping. The model is trained for 300 epochs using stochastic gradient descent with a batch size of 64. The weight decay is set to 1 × $10^{-4}$ , and the momentum at 0.9. The initial learning rate of 0.1 is reduced by a factor of 10 at 50% and 75% of the training process, following a step decay schedule to optimize model convergence.

### 5.2. Experimental Results

#### 5.2.1. Results on KF9

Evaluation of Baseline DCNN Models:

We evaluated the performance of five mainstream CNN models—AlexNet, Inception-Net [26], VGG16, ResNet-101, DenseNet-121—and the ViT [27] model on the KF9 dataset. The results, presented in Table 1, indicate that DenseNet-121 achieved the highest accuracy, while ViT had the lowest. The relatively poor performance of ViT can be attributed to its reliance on large-scale training data to fully leverage its capabilities, which is a limitation when applied to our small-scale KF9 dataset.These findings highlight the strong capability of CNNs in recognizing knitted fabric patterns, with DenseNet-121 excelling in this task. However, due to the predominance of commonly used fabric textures in practical scenarios, the dataset’s scale is somewhat limited by the availability of rare fabric types. Thus, achieving high performance on this dataset is crucial. Based on the optimal CNNs in this comparison, DenseNet, we develop DLW-DenseNet. By integrating weighting mechanisms and differential learning strategies, our model achieve faster convergence and superior performance on the fabric dataset, effectively addressing the dataset’s limitations.

**Figure 8:**
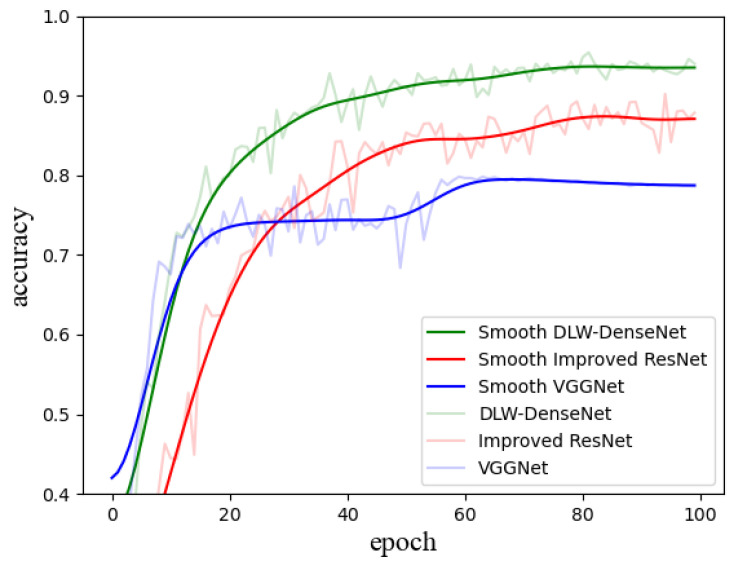
The comparison of performance among the improved ResNet, VGGNet-16, and our proposed network. The horizontal axis represents the number of iterations, while the vertical axis denotes classification accuracy. The solid line represents the smoothed data, whereas the dashed line corresponds to the original data.

Comparison to Previous Deep Learning Fabric texture Recognition Models:

The experimental results on the KF9 dataset, as shown in Figure 8, reveal that our DLW-DenseNet model significantly outperforms other fabric texture recognition convolutional networks. These include the ResNet-based fabric texture recognition network [8] and the VGGNet-based fabric texture recognition network [9]. While VGGNet, with its relatively simple architecture, exhibits rapid accuracy gains in the early stages of training, its performance stagnates in later epochs due to inherent architectural limitations. In contrast, DLW-DenseNet not only surpasses VGGNet but also outperforms the modified ResNet in terms of both training speed and final accuracy. After 100 training epochs, the modified ResNet achieves a peak accuracy of 90.2%, and VGGNet-16 reaches 79.8%. Our DLW-DenseNet, however, achieves a notable 95.4% accuracy, highlighting its superior capability in fabric texture recognition.

The experimental results indicate that our model outperforms other fabric texture recognition convolutional networks in both convergence speed and accuracy, proving the effectiveness of channel weighting and differential learning strategies. Fabric texture recognition is challenging due to the small dataset size and variations in color, yarn diameter, orientation, and lighting conditions. By employing direct channel weighting and differential learning strategies, DLW-DenseNet effectively minimizes redundant information, allowing the model to focus on critical features. This targeted feature selection allows the model to achieve high accuracy, even when trained on a relatively small dataset.

Set the differential learning rate for different multiples:

We systematically tested differential learning rate ratios, where the learning rate for channel weights was set to be 5 to 100 times higher than that of the rest of the model. As shown in Table 2, under our hardware configuration, the model’s performance improved progressively as the ratio increased from 5 to 50 times. However, performance began to decline when the ratio exceeded 50 and reached 100 times. It is worth noting that the optimal differential learning rate ratio likely varies depending on the hardware setup. Therefore, in our experiments, we selected a ratio of 50 times, which delivered the best performance.

#### 5.2.2. Results on CIFAR-10/100

We also demonstrate the effectiveness of the DLW-DenseNet on the CIFAR-10/100 datasets.

As shown in Table 3, the 40-layer DLW-DenseNet achieves an error rate of 4.93% on CIFAR-10, reflecting a 0.43 percentage point improvement over the original DenseNet (5.36%). Moreover, the accuracy of the 40-layer DLW-DenseNet surpasses that of DenseNet by 7.8%, with only a modest 1.4% increase in the number of parameters. Similarly, on CIFAR-10, the 100-layer DLW-DenseNet achieves a 0.22 percentage point increase in accuracy compared to DenseNet, resulting in an overall accuracy improvement of 5.2%.

On the CIFAR-100 dataset, the 40-layer DLW-DenseNet demonstrates a 3.27% accuracy improvement, and the 100-layer version shows a 3.29% enhancement over DenseNet. However, the performance of the 100-layer DLW-DenseNet on CIFAR-100 is somewhat less satisfactory compared to CIFAR-10, likely due to the smaller number of images per category in CIFAR-100, making it more prone to overfitting when trained with larger networks.

Table 3 also provides a comparison with other models on CIFAR-10/100, including ResNet, Swapout, and Pre-ResNet-1001. Our DLW-DenseNet achieved competitive results, outperforming these methods in terms of accuracy, while only increasing a small number of parameters. These findings demonstrate that DLW-DenseNet enhances DenseNet’s optimization capabilities, yielding superior performance with minimal computational overhead.

### 5.3. Ablation Study

In this section, we validate the effectiveness of our model design through a series of ablation experiments. These experiments are conducted on KF9. DenseNet-121 serves as the base architecture for these studies. The ablation process begins by introducing the direct weighting module, which assigns learnable weights to each input feature channel, followed by the application of the differential learning strategy, which enhances channel selection and achieves a pruning effect.

As shown in Table 4, three models were evaluated: Model A represents the original DenseNet architecture, Model B is the Weighted DenseNet with the direct weighting module incorporated, and Model C is the Differential Learning Weighted DenseNet, which introduces the differential learning strategy.

**Figure 9:**
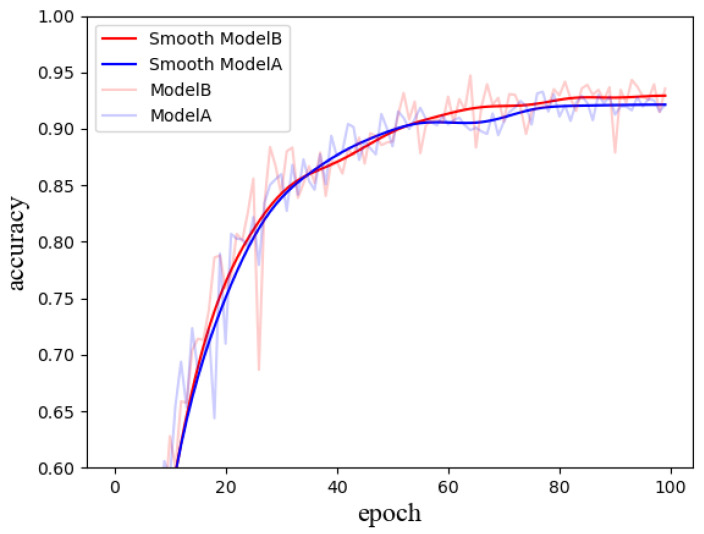
Comparison between the original DenseNet and the Weighted DenseNet.

**Figure 10:**
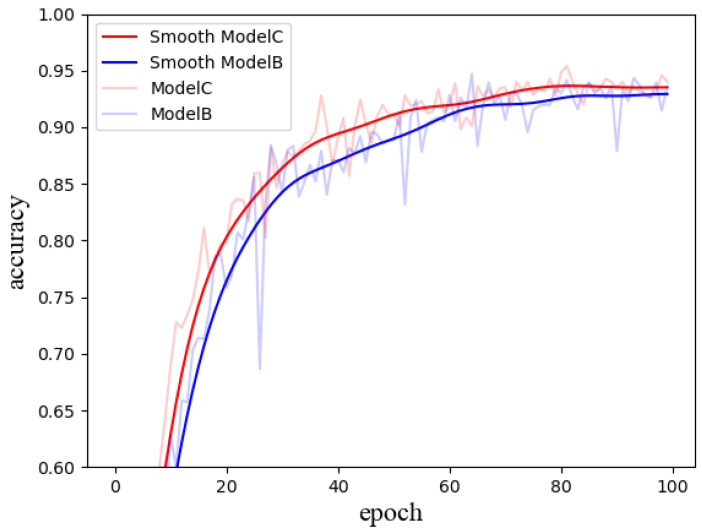
Comparison between the Weighted DenseNet and the DLW-DenseNet.

Figure 9 and Figure 10 illustrate the accuracy comparison between Model A (original DenseNet), Model B (Weighted DenseNet), and Model C (DLW-DenseNet). The shaded regions depict the actual accuracy curves, while the solid lines represent smoothed versions. In Figure 9, the models show substantial oscillation during the initial training phases, with accuracy stabilizing as training progresses. After 100 epochs, Model A reaches an accuracy of 93.2%, while Model B achieves 94.7%, demonstrating a 1.5 percentage point improvement attributed to the direct weighting module’s impact on model performance.

In Figure 10, the differential learning strategy applied in Model C further enhances performance. After 100 epochs, Model C attains an accuracy of 95.4%, which is 0.7 percentage points higher than that of Model B. Additionally, the results indicate that Model C not only surpasses Model B in terms of accuracy but also exhibits faster convergence and greater stability throughout the training process.

## 6. Conclusions

In this paper, we proposed the DLW-DenseNet model for fabric texture recognition, building on the DenseNet architecture. To address challenges such as the limited size of fabric datasets and the effects of variations in position and lighting, we introduced learnable weights for each feature channel and applied a differential learning strategy. Two mechanisms significantly enhanced the model’s ability to capture critical information. Experimental results demonstrated that DLW-DenseNet excels in handling small datasets and exhibits robust resilience to positional and lighting variations. To mitigate the scarcity of fabric datasets, we developed the KF9 knitted fabric dataset, which was used alongside publicly available datasets to train and evaluate models. Results showed that our model outperforms previous state-of-the-art methods in fabric texture recognition tasks and delivers strong results on public datasets. Notably, DLW-DenseNet not only achieves superior accuracy but also operates fewer parameters and reduces computational costs, highlighting its potential for practical applications in the textile and fashion industries. We found that current deep learning-based methods for knitted fabric structure classification are mainly limited to the spatial domain. Compared to texture information, the spatial domain is more suitable for analyzing the structural features of images, whereas the frequency domain can provide a richer and more comprehensive description of texture information. It is better at capturing the fine texture details within the image. Therefore, in the future, we aim to explore the integration of texture information from fabric images for fabric structure classification.

## Figures and Tables

**Table 1:** Performance of different baseline models on the KF9 dataset.

Model.	AlexNet	VGG16	Resnet-101	Inception-Net	DenseNet	VIT
Accuracy	0.869	0.79	0.89	0.91	0.932	0.53

**Table 2:** The effect of differential learning rate multiples on model accuracy.

Difference Multiple	5	10	20	30	40	50	60	70	100
Accuracy (%)	94.85	94.8	95.23	95	95.2	95.4	95.31	95.35	94.9

**Table 3:** Test Error (%) on CIFAR-10/100 by different models.

Model	Parameter	C10	C100
ResNet in ResNet [28]	10.3 M	5.01	22.90
Swapout [29]	7.4 M	4.76	22.72
Pre-ResNet-1001 [30]	10.2 M	4.62	22.71
Densenet-40	1.0592 M	5.36 (5.24 *)	24.73 (24.42 *)
Densenet-100	7.0835 M	4.19 (4.10 *)	20.65 (20.20 *)
DLW-Densenet-40	1.0744 M	4.93	23.92
DLW-Densenet-100	7.1892 M	3.97	19.97

* Note: The results marked with an asterisk are from the original DenseNet [23].

**Table 4:** Differences Between Model A, Model B, and Model C. Model A is the original DenseNet. Model B includes only the direct weighting module. Model C integrates the differential learning strategy along with the direct weighting module.

	Original Densent	Adding Weight	Different Learning
Model A	√	×	×
Model B	√	√	×
Model C	√	√	√

## Data Availability

The original contributions presented in the study are included in the article; further inquiries can be directed to the corresponding author.
