# Correction: Tan et al. An Improved Neural Network Model Based on DenseNet for Fabric Texture Recognition. *Sensors* 2024, *24*, 7758

**DOI:** 10.3390/s25061725

**Published:** 2025-03-11

**Authors:** Li Tan, Qiang Fu, Jing Li

**Affiliations:** College of Science & Technology, Ningbo University, Ningbo 315300, China; 13511146910@163.com (L.T.); lijing3@nbu.edu.cn (J.L.)

## Error in Table

In the original publication [1], the data in Table 1 were erroneously updated prior to the final release owing to confusion with data from other experimental projects. The corrected Table 1 appears below.

We extend our sincere apologies for any issues resulting from the data discrepancies.

The authors state that the scientific conclusions are unaffected. This correction was approved by the Academic Editor. The original publication has also been updated.

## Figures and Tables

**Table 1:** Performance of different baseline models on the KF9 dataset.

Model.	AlexNet	VGG16	Resnet-101	Inception-Net	DenseNet	VIT
Accuracy	0.869	0.79	0.89	0.91	0.932	0.53
